# Nutritional Traits and Physicochemical Parameters of Dry Aged‐In‐Bag and Cooked Serpentina Goat Meat

**DOI:** 10.1002/fsn3.70538

**Published:** 2025-09-23

**Authors:** Menalda V. André, Vítor D. Alves, Catarina Prista, Luísa L. Martins, Mariana Mota, Miguel P. Mourato, André M. de Almeida, Rui J. B. Bessa, Susana P. Alves, Teresa J. S. Matos

**Affiliations:** ^1^ LEAF‐Linking Landscape, Environment Agriculture and Food Research Centre, Instituto Superior de Agronomia Lisboa Portugal; ^2^ Associated Laboratory TERRA Instituto Superior de Agronomia, Universidade de Lisboa Lisboa Portugal; ^3^ CIISA‐Centro de Investigação Interdisciplinar Em Sanidade Animal, Faculdade de Medicina Veterinária Universidade de Lisboa Lisboa Portugal; ^4^ Laboratório Associado Para Ciência Animal e Veterinária (AL4AnimalS) Lisboa Portugal

**Keywords:** cooking process, dry‐aging in bag, goat meat, nutritional traits

## Abstract

*Serpentina* goat is an autochthonous breed from the Alentejo region in Southern Portugal. Meat from kids is a low‐fat protein food that meets consumer demand for healthier and leaner meat options. However, meat from older and heavier animals is less appreciated and often sold at low prices or processed. To promote adult goat meat value, a dry aging process in a bag permeable to water vapor was applied. Effects of aging on cooking loss and of aging and cooking on nutritional properties (minerals, protein, ashes, water content, fatty acids and total fat), pH, color, water activity, nutritional claims, reference intakes and nutrition declaration were investigated. *Longissimus thoracis et lumborum* from five female goats at the end of productive life (8–12 years) were aged 46 days at 2°C ± 2°C and 60%–90% relative humidity. Samples were analyzed after aging, trimming, and cooking in an oven at 150°C until core temperature reached 68°C. Both aging (yield of 72.61% ± 15.2%) and thermal processing led to increased pH values. Aged meat exhibited higher lightness and redness values than regular meat, but cooking increased brightness. In what concerns minerals, potassium content decreased significantly with aging (from 339 to 258 mg/100 g fresh weight), but did not significantly change when cooking the aged meat. The same was observed for magnesium, decreasing only with aging (from 24.7 to 15.2 mg/100 g fresh weight). Minerals like Ca, S and Cu did not change significantly with both processes. A higher concentration of total fatty acids was observed in aged meat, but cooking equalized values between meat samples, with 18:1c9 being the predominant. The n‐6/n‐3 ratio remained within recommended limits (< 4), generally recognized to have a positive impact on the human diet. Regarding nutritional claims, according to the Regulation (EC) n° 1924 (2006), *Serpentina* chevon can be considered as “high protein” and as “low saturated fat” food, with exception for the raw aged meat, slightly above the regulatory limit of 1.5/100 g of meat (1.645/100 g of meat). Both aging and cooking processes affected meat attributes, providing important information to optimize meat quality and consumer demands.

## Graphical abstract

Effects of dry aging‐in‐bag and thermal processes on the nutritional profile of *Serpentina* chevon were investigated. The results revealed significant differences in pH, color, mineral content and fatty acid composition, with the aged and cooked meat showing a profile within the recommended nutritional reference values. Results highlight the potential of dry aging to enhance the underused goat meat, promoting consumer acceptance and increasing nutritional quality.

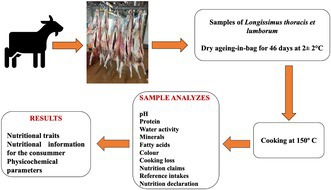

## 1 Introduction

Goat meat (chevon) obtained from kids of the *Serpentina* breed can be certified as *Cabrito do Alentejo*, a European protected geographic indication (PGI) label. The specifications of “Cabrito do Alentejo–PGI” consider goat kids with 30–120 days of age and with carcasses weighing from 3.5 to 7.5 kg. The meat is characterized as lean, exhibiting a light red Hue angle with minimal intensity, tender, juicy and with a pleasant flavor (APCRS 2022). The meat from these younger animals has a remarkable reputation, reaching higher selling prices than that of the non‐certified goat kid meat. Meat from heavier and older animals is not appreciated (Teixeira et al. 2011) in Portugal and is usually sold at low prices and processed into undifferentiated products (Webb et al. 2005). For sustainability and profitability reasons, farmers, meat industrials, and the government are interested in increasing the value of these older animals meat.

The number of registered *Serpentina* breeders is 60, with an inventory of about 6100 female goats (APCRS 2022). Breeding these animals is strategically important for maintaining biodiversity in marginal areas, not only because of their contribution to the cleaning of the bushes and thus the rural fires and desertification prevention in rural interior areas, but also to maintain a unique and very well‐adapted local breed, as well as the human presence in such regions (Di Trana et al. 2015; APCRS 2022; Cerrato et al. 2024).

Meat aging is quite a standard methodology to improve characteristics such as tenderness and flavor in beef (Xu et al. 2021). Different technologies (wet and dry‐aging) have been studied. Dry‐aged meat has more umami than wet‐aged meat (Li et al. 2014). Dry aging is a post‐slaughter procedure in which meat is unpacked and exposed to environmental conditions to improve flavor, tenderness, and product quality. Nevertheless, dry aged meat is more expensive than its fresh counterpart, due to high aging shrinkage, trim loss, risk of contamination, and requirements of aging conditions and space (Parrish et al. 1991). In Portugal, dry‐aged beef typically costs between 50 and 80 €/kg (DRY AGER), while fresh beef round is priced around 13 €/kg (Numbeo 2024) (https://www.numbeo.com). The dry aging process using a high water vapor‐permeable bag (dry‐aging bag) was introduced to the market to improve the traditional unpackaged dry aging process with lower aging and trim losses and similar sensory traits (Ahnström et al. 2006; DeGeer et al. 2009). However, if dry aging of goat meat is not common (Hastie et al. 2021), dry aging in bags is even less. Thus, a critical screening of the dry‐aging in bag process that enables the production of a meat product with higher commercial value and improved nutritional properties from heavier, older, and culled goats from the *Serpentina* breed is necessary and herein proposed.

In this context, an approach was defined to study the processes of dry aging in bag (46 days) and cooking on the nutritional composition (minerals, protein, ash, water content, total fat and fatty acids), pH, cooking loss, and water activity of chevon from the *Serpentina* breed.

## 2 Materials and Methods

### 2.1 Animals and Sampling

**FIGURE 1:**
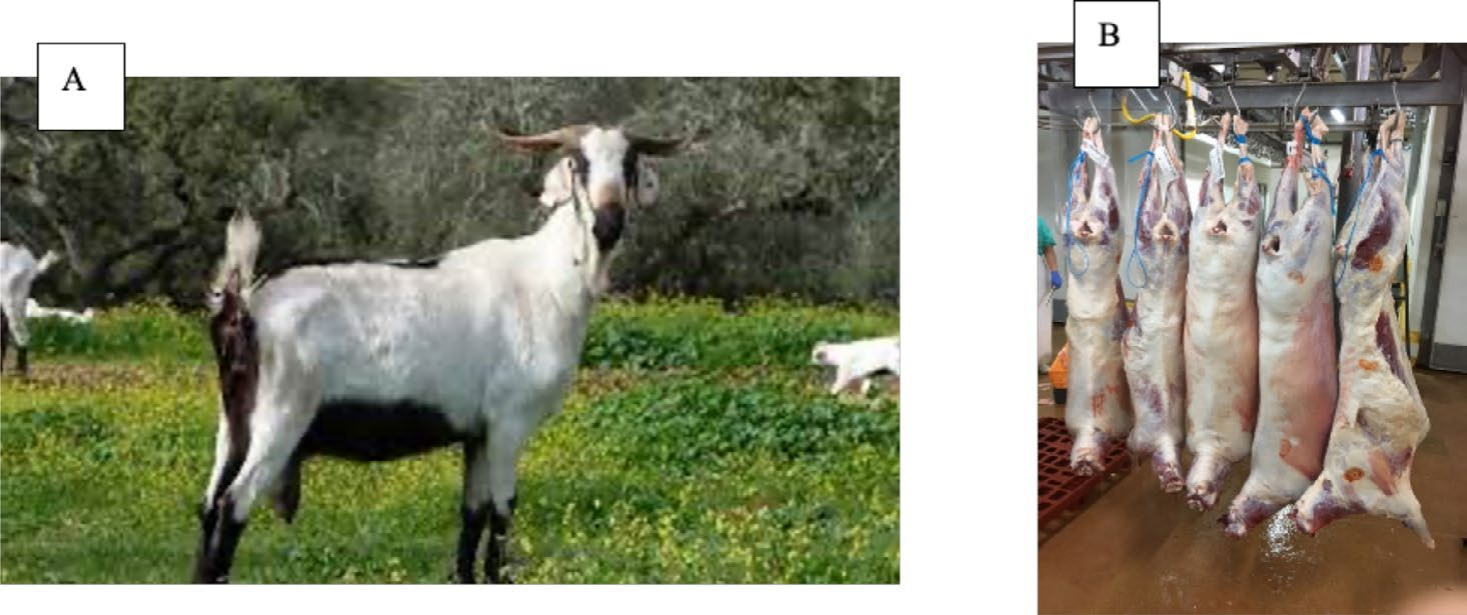
Serpentina goat breed (A: *Serpentina* goat (APCRS 2022); B: Samples of *Serpentina* chevon used in the study).

Five female *Serpentin*a goats at the end of their productive life, aged 8–12 years old, were used in this study. The goats were raised on a commercial farm for the sole purpose of being commercialized for meat production. They were slaughtered on the same day at a commercial abattoir according to standard commercial and regulatory procedures (European Union 2009), subjected to electrical stimulation (330 V, 1.0 A, at 50 Hz for 2 to 3 s) and suspended vertically by hanging through the Achilles tendon, yielding a carcass weight of 28.4 ± 4.4 kg (mean ± standard deviation) (Figure 1). Animal welfare during transport and pre‐slaughter handling was guaranteed in compliance with Council Regulation (EC) n° 1, European Community 2005. On the 2nd day post‐mortem, muscle *Longissimus thoracis et lumborum* (LTL) was cut out from both sides of each carcass and vacuum‐packed. The chilling temperature was 2°C ± 2°C. Five muscle samples from different animals were used, “with the right‐side muscles serving as control and the left‐side muscles assigned to dry aging”.

### 2.2 Aging, Trimming and Sampling

**FIGURE 2:**
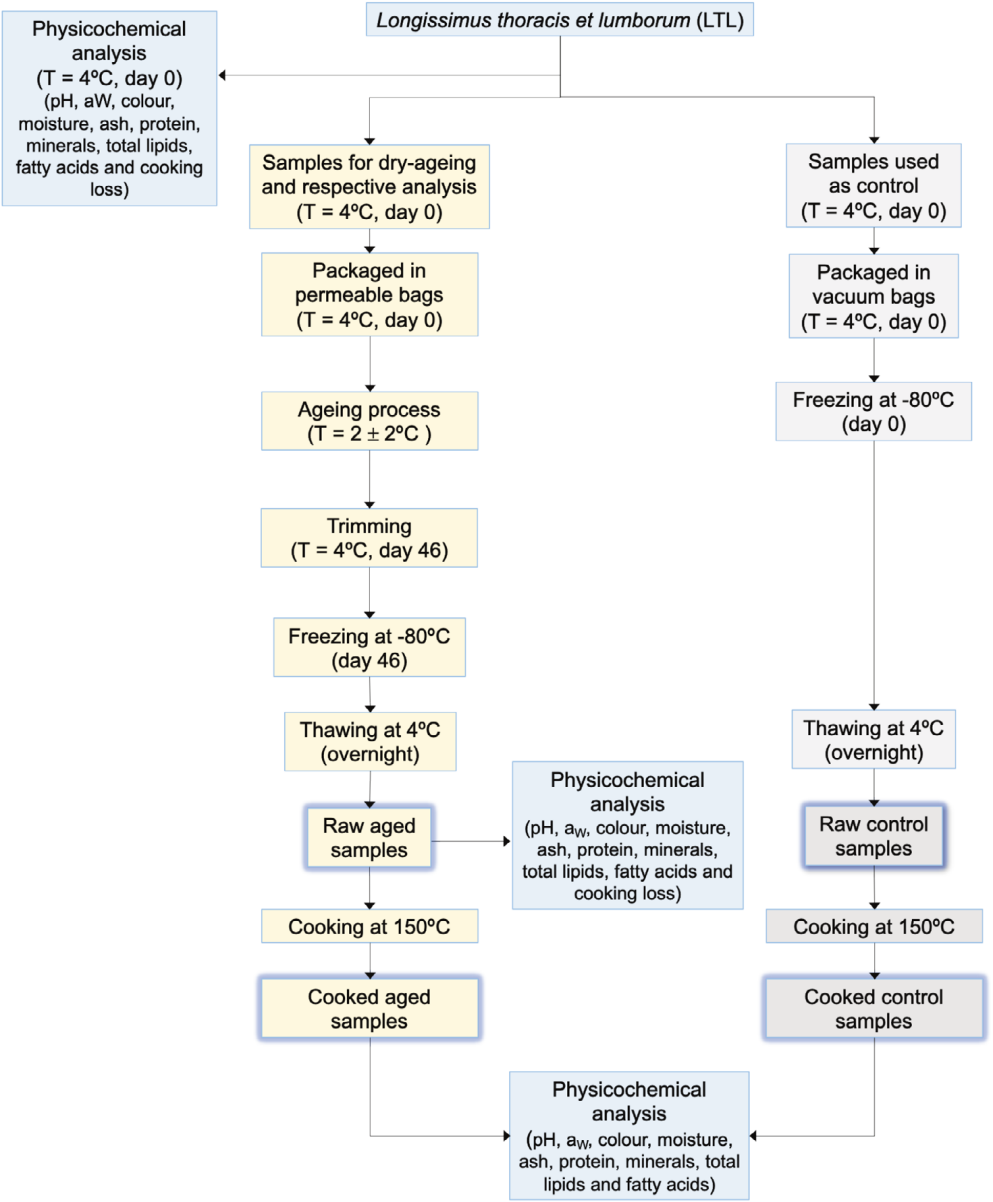
Overall description of the experimental essay involving dry‐aging and cooking at 150°C of Serpentina chevon LTL muscle.

**FIGURE 3:**
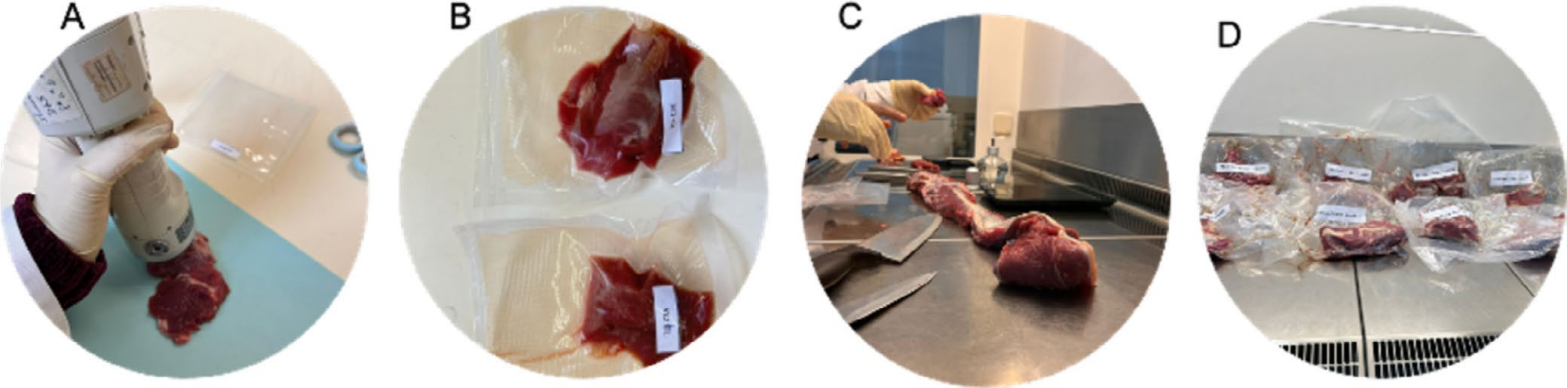
Sample division process (A–D: Color measurements, samples for physicochemical analysis, LTL muscle cut, and samples for dry aging).

On 3rd day post‐mortem samples were separated for physicochemical analysis. Afterwards, five muscle samples of LTL, one from each animal, with an average weight of 1850 ± 429.748 g, were packaged in water vapor permeable bags and subjected to the dry‐aging process (Figure 2; Figure 3). According to the manufacturer, the bags present an O_2_ transmission rate of 24 cm^3^/(m^2^ 24 h) at 23°C and 0% RH; a CO_2_ transmission rate of 78 cm^3^/(m^2^ 24 h) at 23°C and 0% RH; and a water vapor transmission rate of 44 g/(m^2^ 24 h) at 38°C and 100% RH (LID540X, Cryovac, Troisdorf, Germany).

The aging process was carried out at 2°C ± 2°C with relative humidity between 60%–90% for 46 days post‐mortem, in darkness, with non‐filtered air and protected from UV light. At the end of the aging period, samples were trimmed to remove the dry layer on the surface and subjected to physicochemical analysis.

On the same day post‐mortem, five muscle samples of LTL from different animals were kept packaged under vacuum and frozen at −80°C, to be used as control (Figure 2).

The weight losses during aging were calculated as follows:

(1) $$\text{Ageing loss}\;\left(\%\right)=\frac{\text{weight before ageing}-\text{weight after ageing}\;}{\;\text{weight before ageing}}\times 100$$

(2) $$\text{Trimming loss}\;\left(\%\right)=\frac{\text{weight before trimming}-\text{weight after trimming}}{\;\text{weight before trimming}}\times 100$$

(3) $$\text{Ageing and trimming losses}\;\left(\%\right)=\frac{\text{sample weight before ageing}-\text{sample weight after trimming}\;}{\text{sample weight before ageing}}\times 100$$

After separating all samples for analysis at the end of the aging period, the remainder was frozen at −80°C until the cooking process.

The thawing process of aged meat, and non‐aged (control) meat, occurred in a refrigerator at 2°C ± 2°C for 12 h. Afterwards, five samples of raw aged and control meat were simultaneously oven‐roasted in on batch at 150°C until reaching a core temperature of 68°C (around 20 min), and cooled overnight (4°C), after which cooked aged samples and cooked control samples were collected for further analysis.

### 2.3 Cooking Loss of Aged and Non‐Aged Meat

The samples of non‐aged (Day 0, before starting of the aging process) and aged meat (Day 46, at the end of the aging period) were weighed (36.69 ± 4.44 and 43.59 ± 7.65, respectively), vacuum packaged, and heated in a 72°C water bath until reaching a core temperature of 70°C. The samples were cooked in the same batch and then cooled in tap water for 30 min and stored at 4°C overnight. Then, samples were weighed again after opening the package and removing the surface water from the samples (31.73 ± 4.44 and 38.38 ± 7.583, for non‐aged and aged, respectively). Cooking loss was calculated as follows:

(4) $$\text{Cooking loss}\;\left(\%\right)=\frac{\text{weight before heating}-\text{weight after heating}\;}{\text{weight before heating}\;}\times 100$$

### 2.4 Color, Water Activity and pH

Meat color, water activity, and pH were measured in triplicate on the following samples (Figure 2): (i) LTL muscle on the third day post‐mortem, (ii) dry‐aged (Day 46), (iii) dry‐aged (Day 46) and control samples after cooking at 150°C. Meat color (Figure 3) was measured according to the established guidelines by Hunt et al. (2012), using a Konica Minolta CR‐400 (Osaka, Japan), with an aperture size of 3.18 cm and CIE L*, a*, b* color scale. The light source used was the D65 illuminant with a standard observer angle of 10°. After cutting the raw samples to approximately equal sizes, they were allowed to bloom at 4°C for 30 min. The average of nine measurements on the meat surface was used. Chroma (C*) and Hue angle (°h) values were calculated as follows:

(5) $$C^{*}=\sqrt{\left(a^{*2}+b^{*2}\right)}$$

(6)

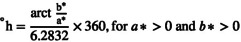




The pH was measured using a portable meter (Crison Instruments, Barcelona, Spain) equipped with a pH electrode suitable for solid products. The pH meter was calibrated with solutions of pH 4 and 7 at room temperature (17°C–20°C) with temperature compensation. Five measurements were performed at different points of each meat sample. Water activity (a_W_) was measured on previously ground samples, using a thermo‐hygrometer at 20°C± 1°C (HigroPalm Rotronic AG, Zurich, Switzerland).

### 2.5 Moisture, Ash, Protein and Mineral Contents

Moisture, ash, protein, and mineral contents were measured using the same samples previously used for pH and color analysis (Figure 3). For meat moisture determination, samples were kept in an oven at 105°C for approximately 24 h, until the percentage difference between two consecutive weighings was below 0.1%. Samples with an initial weight of approximately 2 g were placed in a muffle furnace at 550°C. After 24 h, the final weight of the samples was obtained, and the ash content was determined. Nitrogen content (N) was determined according to the Dumas method from approximately 1 g of fresh biological material, and crude protein content was obtained using the conversion factor of 6.25. To evaluate mineral elements, a mass of samples (around 0.3 g) was digested in 7.5 mL concentrated nitric acid, 2.5 mL concentrated hydrochloric acid, and 1 mL hydrogen peroxide (30%). A subsequent digestion was performed for 2 h at 95°C in an SCP (Science DigiPrep MS digestion system, Jinan, China). Afterwards, the samples were diluted to 25 mL with ultrapure water and analyzed in an ICP‐OES (Thermo Scientific iCAP 7200, Jinan, China) (Ribeiro et al. 2020). Mineral contents were expressed on a fresh weight basis (FW).

### 2.6 Total Lipids and Fatty Acid Composition

Lipids were measured in the same samples indicated in section 2.4. Portions of those samples were separated throughout the essay and immediately frozen at −80°C until lipid analysis. For total lipids and fatty acid analysis, freeze‐dried meat was used for lipid extraction using the (Folch et al. 1957) method, and total lipids were determined gravimetrically. The meat lipid extract was used to prepare the fatty acid methyl esters, as described by Alves et al. (2015). Briefly, the lipid extract was directly trans‐esterified with sodium methoxide 0.5 M in methanol at 50°C for 30 min. This was followed by a reaction with 1.25 M HCL in methanol at 80°C for 15 min. Fatty acids were extracted with hexane, and the excess solvent was removed with a stream of nitrogen at 37°C. The fatty acid extracts were redissolved in 1 mL of hexane and injected onto a gas chromatograph (GC 2010 Plus, Shimadzu, Kyoto, Japan) with a flame ionization detector (FID) and equipped with an SP‐2560 (100 × 0.25 mm, 0.20 μm film thickness, Supelco, Bellefonte, PA, USA) capillary column. The chromatographic conditions were as follows: injector and detector temperatures were set at 250°C and 280°C, respectively; helium was used as the carrier gas at 1 mL/min constant flow; the initial oven temperature of 50°C was held for 1 min, increased at 50°C/min to 150°C and held for 20 min, increased at 1°C/min to 190°C and then increased at 2°C/min to 220°C and held for 30 min. Identification of fatty acid methyl esters was achieved by comparison of their retention times with those of commercial standard mixtures (FAME mix 37 components from Supelco Inc., Bellefonte, PA, USA) and with published chromatograms. Fatty acid results were expressed as milligrams per 100 g of muscle tissue.

### 2.7 Lipid Quality Indexes

The total amount of saturated fatty acids (SFA), monounsaturated fatty acids (MUFA), cis‐monounsaturated fatty acids (cis‐MUFA), trans‐monounsaturated fatty acids (trans‐MUFA), polyunsaturated fatty acids (PUFA), omega‐3 fatty acids (n‐3) and omega‐6 fatty acids (n‐6) were computed and expressed as mg/100 g of muscle. The n‐6/n‐3, MUFA/SFA and PUFA/SFA ratios were also calculated and presented. Additionally, the atherogenicity (AI), and thrombogenicity (TI) indices were computed according to (Ulbricht and Southgate 1991) as follows:

(7) $$\mathrm{AI}=\left(12:0+4\times 14:0+16:0\right)/\left(\sum \text{MUFA}+\sum \text{PUFA}\right)$$

(8) $$\mathrm{TI}=\left(14:0+16:0+18:0\right)/\left(0.5\times \sum \text{MUFA}+0.5\times \sum n-\text{6PUFA}+3\times \sum n-\text{3PUFA}+\left(\sum n-\text{3PUFA}/\sum n-\text{6PUFA}\right)\right)$$

The peroxidizability index (PI) was also calculated (Pamplona et al. 1998):

(9) $$\mathrm{PI}=\left(\%\text{Monoenoic}\times 0.025\right)+\left(\%\text{Dienoic}\times 1\right)+\left(\%\text{Trienoic}\times 2\right)+\left(\text{Tetraenoic}\times 4\right)+\left(\%\text{Pentaenoic}\times 6\right)+\left(\%\text{Hexaenoic}\times 8\right)$$

### 2.8 Nutritional Information for the Consumer

Nutrition declaration, reference intakes, and nutrition claims related to the studied nutrients were determined by applying Regulation (EU) n° 1169 (2011) and Regulation (EC) n° 1924 (2006) instructions. In what concerns nutrition declaration, only minerals with reference intakes ≥ 15% were considered, and salt was calculated as follows (European Union 2011):

(10) $$\text{Salt}=\mathrm{Na}\;\left(\mathrm{mg}\right)\times 2,5$$

Reference intakes were calculated considering an average adult based on 8400 kJ/2000 Kcal according to Regulation (EU) n° 1169 (2011)

(11) $$\text{Refererence intakes}\;\left(\%\right)=\frac{\text{Meat Nutrient quantity}\;\left(g\;\text{or}\;\mathrm{mg}\right)}{\text{Nutrient reference value}\;\left(g\;\text{or}\;\mathrm{mg}\right)}\times 100$$

Nutritional claims designated as high protein and as low saturated fat were calculated according to European Community (2006) guidelines as follows:

- High protein: total meat protein (g) × 4 Kcal ≥ 20% of total meat energy (Kcal).
- Low saturated fat: sum of saturated and trans‐fatty acids ≤ 1.5 g/100 g of meat.

### 2.9 Statistical Analysis

The data were analyzed using the Proc Mixed of SAS 9.4 statistical software (SAS Institute Inc., Cary, NC, USA), with a model that considered the treatments (A—dry‐aging/non‐aging and B—cooking in oven/raw) as fixed effects, following the 2 × 2 factorial treatment arrangement, with dry‐aging and cooking in the oven as main factors and their interaction (A*B). The animal was included in the model as a random block effect. Least square means were calculated, and when an interaction was significant (*p* < 0.05), multiple comparisons of means with Tukey adjustment were conducted. The results are expressed as least square means and standard error of means (*n* = 5).

## 3 Results and Discussion

### 3.1 Process Yield and Cooking Loss

The aging process yield was 72.6%, resulting from a weight loss of 7.58% and a trim loss of 22.0% after 46 days of aging (Table 1). The dry aging process is associated with significant losses due to moisture evaporation (Setyabrata et al. 2022). Previous studies have reported process losses of around 26.7%, 37.1%, and 23.1% for dry‐aged beef in bags for 8, 19, and 21 days, respectively (Bernardo et al. 2021; Li et al. 2014). The aging loss is consistent with that reported by Setyabrata et al. (2022), which obtained a loss of 7.59% after dry aging beef in a bag for 28 days. Furthermore, corroborating this study, it has been reported that weight losses ranging from 5.8% to 13.5%, and trim losses ranging from 22.2% to 27.3% occur in analogous processes (Bernardo et al. 2021).

**TABLE 1:** Effect of dry aging in bag for 46 days on weight and cooking loss. SEM refers to the standard error of means.

Trait	Control non‐aged meat	Dry‐aged in bag	SEM	*p*
Weight loss (%):				
Aging loss	_	7.58	4.81	_
Trim loss	_	22.0	4.15	_
Aging plus trim losses	_	27.4	6.8	_
Cooking loss (%)	2.44	2.07	0.159	0.139

No differences in cooking loss between non‐aged and aged meat were observed (*p* = 0.139; Table 1). Some authors found that aged meat had lower cooking loss than non‐aged due to the low moisture content caused by evaporation during aging (Kim et al. 2020), but in the present case, the moisture content of both non‐aged and aged meat showed no significant differences (Table 2).

**TABLE 2:** Effect of dry aging in bag for 46 days (A) and cooking (B) on proximal and mineral goat meat composition.

Traits	Control		Dry‐aged		SEM	Effects		
	Raw	Cooked	Raw	Cooked		A	B	A × B
Proximal composition (g/100 g fresh weight)								
Moisture	73.9	69.8	74.3	70.7	0.78	0.305	< 0.001	0.664
Protein	22.7	25.5	20.7	25.0	0.65	0.035	< 0.001	0.181
Lipid	2.31^b^	3.75^ab^	4.18^a^	3.33^ab^	0.482	0.071	0.435	0.009
Ashes	1.11	0.97	1.05	1.00	0.022	0.563	< 0.001	0.073
Minerals (mg/100 g fresh weight)								
Na	89.1^a^	64.7^b^	76.3^ab^	69.8^b^	3.59	0.262	< 0.001	0.017
K	339^a^	241^b^	258^b^	265^b^	6.8	< 0.001	< 0.001	< 0.001
Ca	3.80	3.39	1.59	4.33	0.70	0.408	0.142	0.055
Mg	24.7^a^	18.8^b^	15.2^c^	16.7^cb^	0.90	< 0.001	0.008	< 0.001
P	193^a^	173^b^	161^c^	175^b^	2.78	< 0.001	0.329	< 0.001
S	219	251	205	228	92.3	0.011	< 0.001	0.478
Fe	4.40^a^	2.91^ab^	2.66^b^	3.19^ab^	0.39	0.091	0.254	0.025
Cu	0.19	0.35	0.31	0.40	0.02	< 0.001	< 0.001	0.102
Zn	3.83^b^	7.21^a^	6.15^a^	3.12^b^	0.47	0.085	0.720	< 0.001
B	0.14^a^	0.10^b^	0.017^c^	0.086^b^	0.009	< 0.001	0.106	< 0.001
Mn	0.034^a^	0.032^a^	0.022^b^	0.030^ab^	0.002	0.003	0.139	0.023

*Note:* SEM refers to the standard error of means. Means in the same row and with different superscript letters differ significantly (*p* < 0.05).

### 3.2 Color, pH and Water Activity

The color of meat is an important quality parameter that influences consumer purchasing decisions (Mancini and Hunt 2005). Generally, the monochromatic values that determine meat color vary when samples are subjected to aging and thermal processing.

Dry aging in bag and cooking significantly interacted, affecting color parameters (*p* < 0.05) (Table 3). The L* value (luminosity) of raw non‐aged meat was lower (L* = 10.4) than that of raw aged meat. This can be attributed to the higher myoglobin content typically found in the meat of older animals (Neethling et al. 2017), due to increased muscle activity, which in turn contributes to darker meat. Cooking increased the L* value in both types of meat to similarly high levels (≈ 54.1), which may be due to the reduction of myoglobin concentration and water loss (Jiao et al. 2020), possibly as a result of leaching during cooking and heat‐induced myoglobin denaturation. A similar response pattern was also observed for a* and b* color parameters. After aging, the values indicated a lighter meat color (L* = 34.8) and a redder Hue angle (a* = 15.1) (Table 3). These changes were expected after aging, due to water loss, which increases pigment concentration and the denaturation of myofibrillar protein, resulting in a more opaque structure (Holloway and Wu 2019; Zhang et al. 2019). The reason for the lower a* values before aging remains unclear.

**TABLE 3:** Effect of dry aging in bag for 46 days (A) and cooking (B) on pH and water activity (a_W_) and Lab color parameters of goat meat.

Trait	Control		Dry‐aged		SEM^1^	Effects		
	Raw	Cooked	Raw	Cooked		A	B	A × B
pH	5.69^b^	6.49^a^	6.70^a^	6.72^a^	0.138	< 0.001	0.004	0.006
a_W_	0.99^a^	0.87^b^	0.84^c^	0.86^b^	0.005	< 0.001	< 0.001	< 0.001
Color								
L*	10.4^c^	52.9^a^	34.8^b^	55.2^a^	0.95	< 0.001	< 0.001	< 0.001
a*	8.8^c^	17.5^a^	15.1^ab^	14.5^b^	0.72	0.030	< 0.001	< 0.001
b*	9.2^a^	10.8^a^	5.1^b^	10.4^a^	0.42	< 0.001	< 0.001	< 0.001
Croma	26.2	20.6	15.7	17.9	6.61	0.327	0.797	0.553
Hue angle	46.0^a^	32.0^b^	18.6^c^	36.2^b^	1.48	< 0.001	0.258	< 0.001

*Note:* SEM refers to the standard error of means. Means in the same row and with different superscript letters differ significantly (*p* < 0.05).

The interaction between aging and cooking significantly influenced meat color (*p* < 0.001), particularly the a* value. The increase in a* values observed in raw aged meat can be attributed to the reduction in mitochondrial activity in the post‐mortem period, which decreases oxygen consumption and promotes the formation of oxymyoglobin (McKenna et al. 2005). During cooking, despite the denaturation of myoglobin, higher a* values were observed compared to non‐aged raw meat, and were similar to those found in aged raw meat. Although the exact mechanisms behind this phenomenon are not yet fully understood, it is assumed that cooked aged meat tends to maintain higher a* values due to a greater initial proportion of oxymyoglobin and reduced formation of browning pigments such as metmyoglobin. The results therefore suggest that the interaction between aging × cooking can contribute positively to preservation the reddish color of meat.

Cooked meat color varies according to the cooking method and time as thermal processing can alter meat color due to changes in protein structure. In the present study, meat was cooked in the oven at 150°C until the core temperature reached 68°C. Regarding the monochromatic variable b*, which determines the variation from yellow to blue, no significant differences were observed between cooked aged meat and cooked non‐aged meat, showing b* values of around 10 (Table 3). In previous studies (Xiao et al. 2023), b* values around 11 were also reported in raw, non‐aged goat meat frozen at different temperatures. It should be highlighted that in this study, the non‐aged cooked meat was kept frozen at a temperature of −80°C for about 46 days, and the freezing process can affect meat color due to possible protein and pigment degradation. Hue angle value was significantly affected by both aging and cooked processes. The increase in a* value is associated with a decrease in the Hue angle, reflecting a redder hue. This can be attributed to the degradation of myoglobin. In turn, there was no interaction between aging × cooking for Croma (*p* = 0.553).

To the best of our knowledge, the effect of thermal processing on the color and pH of dry‐aged goat meat has not been explored. However, the color of the meat after cooking essentially depends on the modifications of pigments caused by heat, cooking duration, and temperature, primarily associated with pigment denaturation and the decomposition and polymerization of carbohydrates, fats, and proteins (Farraia da Graça 1987).

The pH value is one of the most important indicators of meat quality (Gramatina et al. 2019), as it affects organoleptic characteristics such as tenderness, juiciness, and flavor. Aging and the thermal process, as well as the interaction between them, significantly influenced (*p* = 0.006) the pH values of chevon, with a variation from approximately 5.69 (before aging) to 6.72 (after aging and thermal processing) (Table 3). The significant interaction between aging and thermal processing on pH values can be explained by the effects of proteolysis during aging and protein denaturation during cooking. Aging favors protein degradation and the formation of nitrogen compounds, which tend to increase the pH of meat (Liu et al. 2024); when thermally processed, these aged samples tend to have more protein denaturation than non‐aged samples, resulting in an increase in pH. Other studies (Simela et al. 2004) reported that the pH of goat carcasses varies between 5.8 and 6.2. Huff‐Lonergan (2005) observed that meat pH can vary from approximately 5.2 to 7.0. No significant differences in pH were observed between aged meat and cooked meat samples. Differences in pH values compared to other studies may have arisen from different post‐mortem periods during which pH measurements were taken, although in some studies, the length of the periods was not always well characterized. An increase in pH during aging was reported in dry‐aged lamb meat for 21 days (Zhang et al. 2020). Aging increases meat pH, and this process may be associated with the production of nitrogenous compounds generated by proteolysis (Obuz et al. 2014; Zhang et al. 2019). However, a high pH value (6.7) was reported, suggesting that an alkalization may have occurred during aging, promoting a more tender and juicy meat. This pH value is unfavorable in what concerns meat safety, as it increases the risk of microbial growth of certain pathogens or food spoilage organisms. The optimization of dry aging parameters will be considered, namely aging time and temperature, in order to deliver aged meat with pH values below 6.3.

Dry aging in bag results in a lower water activity (a_W_) compared to conventional wet aging (Li et al. 2014) and, in this case study, the water activity was affected by the aging and cooking processes, decreasing with aging from 0.99 to 0.84 in raw meat and remained unaltered in cooked meat (Table 3). This can be attributed to the bag's permeability, allowing water vapor to escape. Additionally, no significant differences were observed between a_W_ non‐aged and aged cooked meat samples. Water activity is an important parameter for controlling microbial growth in food. Low temperatures combined with reduced a_W_ controls microbial proliferation (Bernardo et al. 2021). However, reports show that low a_W_ reflects the content of free amino acids and aromatic compounds responsible for the development of the characteristic flavor of dry‐aged meat (Ahnström et al. 2006; DeGeer et al. 2009; Li et al. 2014).

### 3.3 Meat Proximal and Mineral Composition

The effects of aging and cooking on protein, ash, moisture, lipids and minerals of meat are presented in Table 2. No interaction between aging × cooking was observed for protein, ashes and moisture. Nevertheless, cooking increased (*p* < 0.001) the protein content and decreased the ash and moisture (*p* < 0.001) independently of aging. Moreover, aging decreased (*p* = 0.035) the protein content independently of cooking, and had no effect on moisture and ash. This increase in protein concentration with cooking can be attributed to a combination of protein denaturation and water loss during thermal processing. In addition, a significant main effect of cooking (*p* < 0.001) on moisture concentration was detected. Thus, cooking led to a lower moisture concentration, with 70.3 ± 0.66 g/100 g compared to 74.1 ± 0.66 g/100 g in the raw meat. Similarly, significant main effects of cooking (*p* < 0.001) were found on the ash content. Here, cooking led to a lower ash concentration, 0.98 ± 0.015 g/100 g compared to 1.08 ± 0.015 g/100 g in the raw meat. In the case of protein, significant main effects were found for aging (*p* = 0.035) and cooking (*p* < 0.001). However, although a lower concentration of protein was observed after aging, with 20.7 g/100 g compared to 22.7 g/100 g in non‐aged meat, these values are quite close to the standard deviation range and may be considered not substantially different. On the other hand, cooking led to higher protein concentration with 25.5 g/100 g compared to 22.7 g/100 g in the raw meat, possibility due to water loss.

A study on the comparison of cooking losses in beef, observed degradation of sarcoplasmic proteins during aging, which may explain these results (Macharáčková et al. 2021). According to the same author (Macharáčková et al. 2021), this phenomenon is attributed to the reduced capacity of the aged muscle to retain water, as breadown of proteins during aging produces smaller fragments that are more easily expelled along with water during heat treatment. In contrast, in non‐aged meat, the water remains more tightly bound to intact, high molecular weight proteins, resulting in lower cooking losses (Macharáčková et al. 2021).

The decrease on ash and moisture with thermal processing can be explained as part of the ash can be lost along with the juices during processing, and moisture loss can occur due to the evaporation of water contained in the meat. Such moisture losses are consistent with the results reported in another study, where dry air thermal processing reduced the moisture content of goat meat (Jiao et al. 2020) and in agreement with other authors (de Lima Cruz et al. 2023) who obtained moisture values between 73.9% and 75% when assessing goat meat quality.

Meat lipid content data presented an interaction between aging × cooking (*p* = 0.009) as the raw non‐aged meat samples presented lower values (2.31/100 g) than raw aged meat (4.18/100 g) and similar intermediate values (≈ 3.5/100 g) when both types of meat were thermally processed (Table 2).

The high lipid content found in raw aged meat may be due to the aging process being done before the removal of subcutaneous fat. As a result, some of the fatty juices may have penetrated the meat during the aging process, which would have increased its lipid content. This might be enhanced due to the fact that they were old goats (8–12 years of age) with good carcass fat cover. Overall, these values are in the same range as those reported by Shija et al. (2013) for goat meat (2.63–4.06/100 g).

The concentration of minerals present in meat is associated with both water and protein contents. In this study, there was an interaction effect between aging and cooking (*p* < 0.05) for most minerals (Na, K, Mg, P, Fe, Zn, Mn and B) (Table 2). On the other hand, for minerals Ca, S, and Cu, there was no interaction between aged and cooked meat (*p* > 0.05) (Table 2).

For Ca, no significant main effects were detected either. However, in the case of S, significant main effects of aging (*p* = 0.011) and cooking (*p* < 0.001) were found. As such, aging led to lower S concentration with 217 ± 8.2 mg/100 g, compared to 235 mg/100 g in non‐aged meat. On the other hand, cooking led to higher S concentration of 240 ± 8.2 mg/100 g, compared to 212 mg/100 g in raw meat. Similarly, for Cu, significant main effects of aging (*p* < 0.001) and of cooking (*p* < 0.001) were found. Here, aging led to higher Cu concentration with 0.36 ± 0.014 mg/100 g, compared to 0.27 mg/100 g in non‐aged meat. Cooking also led to higher Cu concentration with 0.38 ± 0.014 mg/100 g, compared to 0.25 mg/100 g in raw meat.

However, samples of aged raw meat showed lower values of minerals K, Mg, P, Fe, Mn and B compared to non‐aged raw meat, and similar intermediate values when both types of meat were cooked. On the other hand, Jiao et al. (2020) observed an increase in the values of Ca, Mg, and Fe in a study related with the nutritional and safety characteristics of goat meat subjected to thermal processing. The variability observed in minerals concentrations of aged and cooked samples can also be explained by complex factors that increase the solubility and availability of some minerals, while others may be lost during cooking along with the water.

### 3.4 Meat Fatty Acid Composition

Total fatty acid content was affected by the aging × cooking interaction, showing a similar pattern to that described for the meat lipid content as discussed above. Forty‐three fatty acids were identified in the LTL muscle. Among these, 16 saturated fatty acids (SFA), 11 cis monounsaturated fatty acids (cis‐MUFA), six trans monounsaturated fatty acids (trans‐MUFA) and 10 polyunsaturated fatty acids (PUFA) were found. Of these 43 fatty acids, only myristic (14:0), arachidonic (20:4n‐6), and the other 4 minor fatty acids (i.e., 16:1c7, 18:1t9, 18:c15, 22:4n‐6) presented a significant aging × cooking interaction. Aging increased (*p* = 0.052) the 16:0 and decreased (*p* < 0.05) the 18:2n‐6, 18:3n‐6, 18:3n‐3, 20:2n‐6, 18:3c9t11c15, and 20:5n‐3. Cooking increased (*p* < 0.05) 14:1c9, 16:1c9, and 17:1c9 and decreased (*p* = 0.038) the 10:0.

The results of the total meat fatty acids and of the main and nutritional relevant fatty acids are presented expressed in mg per 100 g muscle tissue (Table 4). The total fatty acid contents differ widely between non‐aged and aged raw meat (1.5 vs. 3.4/100 g of meat, *p* = 0.082), but with cooking this effect of meat aging disappears (2.9 vs. 2.3/100 g of non‐aged or aged meat samples, *p* = 0.650). This clear interaction between dry aging and cooking on total fatty acid content shows a similar pattern to that described for the meat lipid content, as discussed above. The content of most of the major or nutritionally relevant meat fatty acids also presents a similar aging × cooking interaction response pattern (*p* < 0.05) as it presents significantly lower content of the major fatty acids and major partial sums (i.e., 16:0, 18:0, 18:1c9, SFA, *cis*‐MUFA, *trans*‐MUFA and PUFA) in raw non‐aged meat than in raw dry aged meat. The effect of cooking is more variable as decreased the 18:0, 18:2n‐6, 18:3n‐3, SFA, trans‐MUFA and PUFA in dry‐aged samples and or no effect or even increase (e.g., 18:3n‐3, 18:2c9t11 and total PUFA) in non‐aged samples. As all these differences are the reflex of the large variations observed for total fatty acid and lipid content, it is useful to interpret the fatty acid profile expressed as the percentage of total fatty acids. Thus, the detailed fatty acid composition of meat samples is presented in Table 5.

**TABLE 4:** Effect of dry aging in bag for 46 days (A) and cooking (B) on main fatty acid content (mg/100 g muscle tissue) of goat meat.

Traits	Control		Dry‐aged		SEM*	Effects		
	Raw	Cooked	Raw	Cooked		A	B	A × B
Total FA	1536^b^	2878^ab^	3360^a^	2333^ab^	337	0.082	0.650	0.004
Fatty acids								
16:0	386^b^	589^ab^	788^a^	507^ab^	74.1	0.052	0.608	0.007
16:1*c*9	27	62	54	46	8.7	0.546	0.150	0.028
18:0	300^b^	478^ab^	703^a^	388^b^	68.1	0.041	0.334	0.004
18:1*c*9	634^b^	1022^ab^	1217^a^	835^ab^	126	0.144	0.982	0.010
18:2n‐6	51^ab^	69^a^	70^a^	48^b^	5.0	0.836	0.665	0.001
18:3n‐3	16^c^	23^ab^	25^a^	17^bc^	1.5	0.365	0.541	< 0.001
18:2*c*9*t*11	3.8^b^	7.6^a^	8.7^a^	5.9^ab^	0.83	0.086	0.576	0.002
20:4n‐6	26	27	24	22	1.07	0.003	0.808	0.122
20:5n‐3	9.7	9.5	8.6	8.2	0.41	0.014	0.500	0.733
22:5n‐3	13^ab^	16^a^	14^ab^	12^b^	0.65	0.093	0.665	0.001
22:6n‐3	1.8	2.0	1.8	1.5	0.16	0.174	0.716	0.257
Partial Sums								
SFA	752^b^	1199^ab^	1645^a^	991^b^	153	0.046	0.513	0.004
*cis*‐MUFA	691^b^	1149^ab^	1330^a^	924^ab^	139	0.164	0.855	0.009
*trans*‐MUFA	19^b^	33^ab^	47^a^	26^b^	4.38	0.030	0.481	0.002
Total PUFA	129^b^	164^a^	162^a^	121^b^	7.5	0.499	0.681	< 0.001
n‐3	40^b^	50^a^	50^a^	38^b^	1.760	0.502	0.591	< 0.001
n‐6	81^ab^	101^a^	99^a^	73^b^	5.78	0.376	0.655	0.001
Ratios and Indexes								
n‐6/n‐3	2.01	2.03	1.99	1.89	0.174	0.496	0.736	0.579
MUFA/SFA	0.93	1.01	1.08	0.94	0.049	0.212	0.115	0.945
PUFA/SFA	0.19	0.16	0.86	0.94	0.018	0.009	0.888	0.130
AI**	0.60	0.60	0.69	0.65	0.021	0.008	0.291	0.300
TI***	1.37	1.38	1.75	1.51	0.075	0.005	0.148	0.110
PI****	26.4^a^	20.5^ab^	14.2^b^	18.3^ab^	0.09	0.005	0.673	0.035

* SEM refers to the standard error of means. Means in the same row and with different superscript letters differ significantly (*p* < 0.05).

** Atherogenicity index.

*** Thrombogenicity index.

**** Peroxidizability index.

**TABLE 5:** Effect of dry aging in bag for 46 days (A) and cooking (B) on detailed fatty acid profile (% of total fatty acids) of goat meat.

Traits	Control		Dry‐aged		SEM	Effects		
	Raw	Cooked	Raw	Cooked		A	B	A × B
10:0	0.10	0.10	0.11	0.09	0.006	0.306	0.038	0.237
12:0	0.07	0.09	0.08	0.07	0.006	0.975	0.332	0.053
i‐14:0	0.03	0.04	0.04	0.04	0.005	0.243	0.761	0.129
14:0	1.90^b^	2.25^a^	2.17^ab^	2.17^ab^	0.071	0.197	0.029	0.031
i‐15:0	0.09	0.21	0.13	0.14	0.036	0.593	0.091	0.132
a‐15:0	0.08	0.12	0.13	0.10	0.017	0.424	0.771	0.146
14:1*c*9	0.08	0.21	0.08	0.13	0.038	0.341	0.040	0.326
15:0	0.29	0.53	0.36	0.37	0.073	0.540	0.129	0.141
i‐16:0	0.13	0.21	0.16	0.15	0.023	0.690	0.151	0.077
16:0	24.15	23.01	24.64	24.57	0.304	0.006	0.073	0.105
i‐17:0	0.29	0.36	0.33	0.31	0.034	0.911	0.421	0.230
16:1*c*7	0.36^a^	0.42^a^	0.42^a^	0.38^a^	0.020	0.661	0.577	0.028
16:1*c*9	1.65	2.45	1.72	2.14	0.217	0.589	0.016	0.409
a‐17:0	0.26	0.22	0.21	0.22	0.037	0.528	0.658	0.456
17:0	0.78	0.94	0.86	0.81	0.065	0.729	0.419	0.131
i‐18:0	0.09	0.11	0.10	0.10	0.008	0.892	0.403	0.550
17:1*c*9	0.50	0.91	0.47	0.60	0.106	0.145	0.026	0.205
18:0	18.94	18.27	21.91	19.13	0.937	0.063	0.091	0.279
18:1 *t*6/*t*7/*t*8	0.12	0.13	0.14	0.14	0.010	0.322	0.687	0.459
18:1 *t*9	0.16^b^	0.19^a^	0.20^a^	0.18^ab^	0.008	0.061	0.187	0.010
18:1 *t*10	0.11	0.09	0.14	0.10	0.017	0.236	0.164	0.522
18:1 *t*11	0.61	0.64	0.76	0.70	0.114	0.373	0.898	0.689
18:1 *t*12	0.13	0.12	0.11	0.11	0.011	0.205	0.817	0.568
18:1*c*9	38.86	39.74	38.23	39.69	1.384	0.809	0.416	0.839
18:1*c*11	0.88	0.95	0.76	0.85	0.063	0.113	0.217	0.894
18:1*c*12	0.08	0.07	0.08	0.07	0.005	0.877	0.139	0.444
18:1*c*13	0.03	0.05	0.03	0.04	0.011	0.374	0.162	0.484
18:1*c*14	0.11	0.13	0.15	0.13	0.010	0.069	0.586	0.060
18:1*c*15	0.028^b^	0.034^ab^	0.041^a^	0.032^ab^	0.003	0.048	0.568	0.019
18:2n‐6	3.45	2.99	2.28	2.42	0.235	0.003	0.504	0.224
20:0	0.07	0.09	0.10	0.08	0.010	0.377	0.864	0.132
18:3n‐6	0.028	0.024	0.019	0.020	0.001	< 0.001	0.332	0.076
18:3n‐3	1.11	0.96	0.82	0.86	0.084	0.037	0.573	0.295
18:2*c*9*t*11	0.25	0.31	0.29	0.30	0.038	0.849	0.378	0.542
20:2n‐6	0.03	0.02	0.02	0.02	0.003	0.043	0.817	0.138
18:3*c*9*t*11*c*15	0.32	0.25	0.17	0.22	0.035	0.025	0.732	0.098
22:0	0.03	0.02	0.02	0.021	0.003	0.159	0.975	0.476
20:3n‐6	0.02	0.12	0.08	0.11	0.025	0.092	0.878	0.146
20:4n‐6	1.85^a^	1.28^ab^	0.79^b^	1.16^ab^	0.207	0.015	0.648	0.041
20:5n‐3	0.71	0.46	0.29	0.45	0.097	0.048	0.658	0.057
22:4n‐6	0.07^a^	0.06^ab^	0.04^b^	0.05^ab^	0.005	0.004	0.673	0.011
22:5n‐3	0.90	0.74	0.48	0.63	0.138	0.082	0.989	0.282
22:6n‐3	0.13	0.10	0.06	0.08	0.024	0.098	0.848	0.252
Partial sums								
SFA	47.3	46.6	51.3	48.4	1.15	0.025	0.134	0.351
*cis*‐MUFA	42.5	44.8	41.8	43.9	1.51	0.620	0.166	0.929
*trans*‐MUFA	1.24	1.31	1.50	1.36	0.128	0.242	0.796	0.435
Total PUFA	9.01	7.31	5.32	6.34	0.84	0.017	0.689	0.132
n‐3 PUFA	2.84	2.26	1.65	2.03	0.364	0.056	0.774	0.181
n‐6 PUFA	5.59	4.49	3.22	3.79	0.59	0.008	0.586	0.105

*Note:* SEM refers to standard error of means. Means in the same row and with different superscript letters differ significantly (*P* < 0.05).

The partial sums of fatty acids expressed as percentage of total fatty acids (Table 5) were not affected by cooking or aging × cooking interaction. However, aging increased (*p* = 0.025) SFA and decreased (*p* = 0.017) PUFA, particularly the n‐6 PUFA (*p* = 0.008), which is consistent with the effects detected in 14:0, 16:0, 20:4n‐6 and other minor PUFA. The explanation of the increase in SFA and decrease in PUFA with aging is probably due to the infiltration of more saturated fatty acids from lipolysis of adipose tissue with dilution of the muscle membrane lipids rich in PUFA, and also as result of lipid peroxidation during the long aging period. Lean meat is especially rich in phospholipids, which contain membrane long‐chain PUFA, specifically 20:4n‐6. On the other hand, meat with a high intermuscular and subcutaneous fat content is comprised mostly of triacylglycerols rich in SFA and MUFA and a lower content of long‐chain PUFA (Bessa et al. 2015). Losses of PUFA in aged meat due to peroxidation have been reported (Holman et al. 2019; Mahecha et al. 2009).

The SFA presented the highest proportion (46.6%–51.3%), followed by MUFA (43.3%–46.2%), and PUFA with 5.3%–9.0%. A predominance of SFA and a lower presence of PUFA is often reported in goat meat (Madruga and Bressan 2011; Talpur et al. 2008). The most abundant SFA are 16:0 (23.0%–24.6%) and 18:0 (18.3%–21.9%) which is what is expected in ruminant meat (Vahmani et al. 2020). The 16:0 contents observed in this study were within the range of values previously reported on beef aging (Di Paolo et al. 2023), as well as for dry‐aged and wet‐aged beef for 35 days (Mikami et al. 2021).

The 18:2c9t11, the main conjugated isomer of linoleic acid (CLA), has interesting biological actions including anticarcinogenic activity (Dilzer and Park 2012; Park 2009). It was not affected by treatments and was present around 0.25%–0.31%, which is the usual in most ruminant meat (Bessa et al. 2015; Di Paolo et al. 2023; Talpur et al. 2008).

The MUFA comprise on average, 44.7% of fatty acids, with 18:1c9 being the most prominent, making up around 86% of the total MUFA. This is what is commonly expected in ruminant meat (Vahmani et al. 2020). The 18:1c9 and MUFA were not affected by treatments; however, cooking increased the proportions of some minor cis‐MUFA, as the 14:1c9, 16:1c9 and 17:1c9. The reasons for that increase are not clear to us.

The ratios and nutritional relevant fatty acid indices are presented in Table 4. The n‐6/n‐3 PUFA ratio was not affected by treatments. Dry‐aging increased the PUFA/SFA ratio (0.18 vs. 0.90, *p* = 0.009), the AI (0.60 vs. 0.68, *p* = 0.008) and the TI (1.38 vs. 1.63, *p* = 0.005). The cooking had no effect on these variables. The TI values reported by us are within the ranges found by Di Paolo et al. (2023). These results are consistent with the findings that the dry‐aging process increased SFA and decreased PUFA, as discussed previously. The AI results are consistent with the differences observed in the lipid and fatty acid content. AI and TI indexes are linked to the impact of fatty acids on human health (Pretorius and Schönfeldt 2021) and Ulbricht and Southgate (1991) proposed that sheep meat should have AI and TI values of up to 1.0 and 1.58, respectively. Thus, lower AI and TI values indicate higher proportions of anti‐atherogenic fatty acids in the lipid fraction, potentially reducing the risk of coronary and cardiovascular diseases (Ulbricht and Southgate 1991).

The PI was higher in non‐aged raw meat and decreased with aging, while cooked meat from both treatments showed similar intermediate values (aging × cooking, *p* = 0.004). Increased PI is considered beneficial as it is positively associated with the nutritional quality of meat. However, at high levels, meat may be susceptible to oxidation.

### 3.5 Nutritional Information for the Consumer

Goat meat is recognized as an important source of essential nutrients for the human body. However, the effects of dry aging in bag and of cooking on the nutritional composition of this meat have not yet been extensively studied. Table 6 presents the nutritional declaration with the reference values of energy, protein, total fat, saturates, mono and polyunsaturated fatty acids, salt, and minerals (P, Fe, Zn and Cu) with reference intakes ≥ 15%, determined in the LTL muscle of aged and cooked *Serpentina* chevon. The results indicated that the consumption of 100 g of this cooked and previously aged meat provides 6.53% of the recommended daily energy value for an average adult, based on a diet of 2000 kcal/day. The total fat, protein, and mineral values represent the average obtained in both treatments.

**TABLE 6:** Nutrition declaration per 100 g of goat meat (average values; *n* = 5).

Goat meat	Control				Dry‐aged			
	Raw		Cooked		Raw		Cooked	

%RI*		%RI*		%RI*		%RI*
Energy**	471.37 kJ 111.59Kcal	5.61^a^ 5.58^a^	572.25 kJ 135.75Kcal	6.81^a^ 6.70^a^	506.56 kJ 120.42Kcal	6.03^a^ 6.02^a^	548.21 kJ 129.97Kcal	6.53^a^ 6.50^a^
Total fat (g)	2.31	3.30^b^	3.75	5.36^b^	4.18	5.97^b^	3.33	4.76^b^
Saturates (g)	0.752	3.76^c^	1.199	5.99^c^	1.645	8.23^c^	0.991	4.96^c^
Mono‐unsaturates (g)	0.71		1.182		1.377		0.95	
Polyunsaturates (g)	0.129		0.164		0.162		0.121	
Protein (g)	22.71	45.42^d^	25.5	51^d^	20.7	41.4^d^	25	50^d^
Salt (g)***	0.22	3.67^e^	0.16	2.67^e^	0,19	3.17^e^	0,17	2.83^e^
P (mg)	193	27.57^f^	173	24.71^f^	161	23^f^	175	25^f^
Fe (mg)	4.4	31.43^g^	2.91	20.79^g^	2.66	19^g^	3.19	22.79^g^
Zn (mg)	3.83	38.3^h^	7.21	72.1^h^	6.15	61.5^h^	3.12	31.2^h^
Cu (mg)	0.19	19^i^	0.35	35^i^	0.31	31^i^	0.4	40^i^

* RI: reference intake with reference doses for an average adult (8400 kJ/2000 Kcal) (Regulation (EU) n° 1169, 2011). %RI calculations were performed considering the dietary reference intakes presented in attachment XIII of the Regulation (EU) n° 1169 (2011), as follows for each letter of Table 6.

** Energy was calculated according to Regulation (EU) n°1169/2011 (Energy: 1 g of Protein = 17 kJ/g and 4 Kcal/g and 1 g of Lipid = 37 kJ/g and 9 Kcal/g).

*** Salt was calculated according to Regulation (EU) n°1169/2011 (Salt: Na (g) × 2.5). Salt content is exclusively due to the presence of naturally occurring Na.

^a^ %RI of energy for goat meat (control) = (471.37/8400 kJ) × 100 = 5.61% (Raw) and (572.25/8400 kJ) × 100 = 6.81% (Cooked); (111.59/2000 Kcal) × 100 = 5.58% (Raw) and (135.75/2000 Kcal) × 100 = 6.70% (Cooked) and %RI of energy for goat meat (dry‐aged) = (506.56/8400 kJ) × 100 = 6.03% (Raw) and (548.21/8400 kJ) × 100 = 6.53% (Cooked); (120.42/2000 Kcal) × 100 = 6.02% (Raw) and (129.97/2000 Kcal) × 100 = 6.50% (Cooked).

^b^ %RI of Total fat for goat meat (control) = (2.31/70 g) × 100 = 3.30% (Raw) and (3.75/70 g) × 100 = 5.36% (Cooked); %RI of Total fat for goat meat (dry‐aged) = (4.18/70 g) × 100 = 5.97% (Raw) and (3.33/70 g) × 100 = 4.76% (Cooked).

^c^ %RI of Saturates for goat meat (control) = (0.752/20 g) × 100 = 3.76% (Raw) and (1.199/20 g) × 100 = 5.99% (Cooked); %RI of Saturates for goat meat (dry‐aged) = (1.645/20 g) × 100 = 8.23% (Raw) and (0.991/20 g) × 100 = 4.96% (Cooked).

^d^ %RI of Protein for goat meat (control) = (22.71/50 g) × 100 = 45.42% (Raw) and (25.5/50 g) × 100 = 51% (Cooked); %RI of Protein for goat meat (dry‐aged) = (20.7/50 g) × 100 = 41.4% (Raw) and (25/50 g) × 100 = 50% (Cooked).

^e^ %RI of Salt for goat meat (control) = (0.22/6 g) × 100 = 3.67% (Raw) and (0.16/6 g) × 100 = 2.67% (Cooked); %RI of Salt for goat meat (dry‐aged) = (0.19/6 g) × 100 = 3.17% (Raw) and (0.17/6 g) × 100 = 2.83% (Cooked).

^f^ %RI of P for goat meat (control) = (193/700 mg) × 100 = 27.57% (Raw) and (173/700 mg) × 100 = 24.71% (Cooked); %RI of P for goat meat (dry‐aged) = (161/700 mg) × 100 = 23% (Raw) and (175/700 mg) × 100 = 25% (Cooked).

^g^ %RI of Fe for goat meat (control) = (4.4/14 mg) × 100 = 31.43% (Raw) and (2.91/14 mg) × 100 = 20.79% (Cooked); %RI of Fe for goat meat (dry‐aged) = (2.66/14 mg) × 100 = 19% (Raw) and (3.19/14 mg) × 100 = 22.79% (Cooked).

^h^ %RI of Zn for goat meat (control) = (3.83/10 mg) × 100 = 38.3% (Raw) and (7.21/10 mg) × 100 = 72.1% (Cooked); %RI of Zn for goat meat (dry‐aged) = (6.15/10 mg) × 100 = 61.5% (Raw) and (3.12/10 mg) × 100 = 31.2% (Cooked).

^i^ %RI of Cu for goat meat (control) = (0.19/1 mg) × 100 = 19% (Raw) and (0.35/1 mg) × 100 = 35% (Cooked); %RI of Cu for goat meat (dry‐aged) = (0.31/1 mg) × 100 = 31% (Raw) and (0.4/1 mg) × 100 = 40% (Cooked).

According to the guidelines established by the Regulation (EU) n°1169 (2011) the recommended daily protein intake for adults is 50 g. Our analysis revealed that the consumption of a 100 g portion of goat meat, which had been aged and then cooked, provides 41.4% and 50% of this reference value, respectively (Table 6). It is interesting to note that the control samples, which did not go through the aging process but were cooked, had an even more significant protein contribution, reaching 51% respectively of the daily requirement (Table 6). The data suggests that heat treatment increases the accessibility of proteins, since before cooking, the contribution for the reference value was only 45%. This observation is consistent with recent research, which demonstrated that culinary heat treatment significantly increases protein density in meat, attributable to substantial moisture loss (Ruxton and Gordon 2024).

With regard to the daily intake of fats, Regulation (EU) n°1169 (2011) recommends a reference value of 70 g for an average adult, considering a diet of 2000 Kcal/day. Our research has shown that eating a 100 g portion of goat meat, which has been aged and then cooked, provides between 3.3% and 4.76% of the recommended daily amount (Table 6). In addition, the same regulation suggests a limit of 20 g for daily consumption of saturated fatty acids. In this context, we found that the same portion of processed goat meat represents between 3.76% and 8.23% of this reference value.

The micronutrient analysis, carried out in accordance with parameters established by the Regulation (EU) n°1169 (2011), showed that the levels of Fe, Zn, P and Cu in *Serpentina* chevon, regardless of the treatment applied, exceeded 15% of the daily reference values, as shown in Table 6. Of particular note is the significant contribution of Zn in both treatments. This mineral contributes significantly more than the other minerals analyzed, with a range from 31% to 72% of the daily reference value, depending on the applied treatment.

In the context of nutritional claims, according to Regulation (EC) n° 1924 (2006) *Serpentina* chevon is considered “high protein content” as it has a protein content which provides more than 20% of the total meat energy value, irrespective of aging. In addition, this meat has a low saturated fat content in most of the treatments analyzed (< 1.5/100 g of meat). However, the raw aged meat is an exception, with a saturated fat content of 1.645 g per 100 g, slightly above the regulatory limit of 1.5 g. Nutritional claims reported for goat meat in this manuscript (established accordingly to Regulation (EC) n° 1924, 2006), such as “high protein content” and “low saturated fat content” could be compared with other types of meat through their composition in energy (Kcal/100 g of meat), in protein (g/100 g of meat) contents and determining the protein contribution for total meat energy (if protein content (g/100 g of meat) × 4 Kcal/g of protein > 20% of total meat energy (Kcal)/100 g of meat) and also, through their content of total saturated fatty acids (g) per 100 g of meat (if < 1.5 g of saturated fatty acids/100 g of meat). Normally, beef and pork present values for saturated fatty acids between 1.4–4.1/100 g of meat, accordingly food composition tables of the National Institute of Health Doctor Ricardo Jorge of the Portuguese Ministry of Health (INSA 2023) (https://portfir‐insa.min‐saude.pt/foodcomp/search), which shows that goat meat could be more healthy than meat from other animal species.

## 4 Conclusions

Aging resulted in a lighter meat color with a more pronounced reddish Hue angle and thermal processing influenced meat color by increasing lightness and slightly decreasing redness values. These changes could be attributed to heat‐induced modifications of meat pigments and proteins. Aging did not influence meat protein levels, but cooking led to an increase in protein concentration, possibly associated with water loss. These factors could also influence the observed results in the contents of certain minerals. The study also revealed that pH values increased during the aging process. Regarding fatty acids, the aging process increased the SFA and reduced the PUFA in meat. The impact of cooking on fatty acids was negligible in aged meat. The analysis of the nutritional profile of goat meat revealed that the aging and cooking process have a positive influence on its composition. The application of these processing tecniques results in meat closer to the recommended nutritional regulations.

Overall, the study sheds light on the effects of aging and cooking processes on various parameters related to goat meat quality, providing valuable information for both consumers and meat industry. Further research is needed to explore overall consumer acceptability of aged *Serpentina* chevon.

## Author Contributions

**Menalda V. André:** data curation (equal), formal analysis (equal), investigation (equal), visualization (equal), writing – original draft (equal), writing – review and editing (equal). **Vítor D. Alves:** conceptualization (equal), formal analysis (equal), investigation (equal), methodology (equal), supervision (equal), validation (equal), visualization (equal), writing – original draft (equal), writing – review and editing (equal). **Catarina Prista:** conceptualization (equal), formal analysis (equal), investigation (equal), methodology (equal), resources (equal), supervision (equal), visualization (equal), writing – original draft (equal), writing – review and editing (equal). **Luísa L. Martins:** formal analysis (equal), writing – original draft (equal), writing – review and editing (equal). **Mariana Mota:** data curation (equal), formal analysis (equal), writing – original draft (equal), writing – review and editing (equal). **Miguel P. Mourato:** formal analysis (equal), writing – original draft (equal), writing – review and editing (equal). **André M. de Almeida:** conceptualization (equal), formal analysis (equal), writing – original draft (equal), writing – review and editing (equal). **Rui J. B. Bessa:** data curation (equal), formal analysis (equal), writing – original draft (equal), writing – review and editing (equal). **Susana P. Alves:** data curation (equal), formal analysis (equal), writing – original draft (equal), writing – review and editing (equal). **Teresa J. S. Matos:** conceptualization (equal), data curation (equal), formal analysis (equal), funding acquisition (equal), investigation (equal), methodology (equal), project administration (equal), supervision (equal), validation (equal), writing – original draft (equal), writing – review and editing (equal).

## Conflicts of Interest

The authors declare no conflicts of interest.

## Data Availability

The data that support the findings of this study are available on request from the corresponding author.
